# Tebipenem as an oral alternative for the treatment of typhoid caused by XDR *Salmonella* Typhi

**DOI:** 10.1093/jac/dkab326

**Published:** 2021-09-17

**Authors:** Elli Mylona, Phat Voong Vinh, Sonia Qureshi, Abhilasha Karkey, Sabina Dongol, Tuyen Ha Thanh, Judd Walson, Lluis Ballell, Elena Fernández Álvaro, Farah Qamar, Stephen Baker

**Affiliations:** 1 University of Cambridge School of Clinical Medicine, Cambridge Biomedical Campus, Cambridge, UK; 2 Department of Medicine, University of Cambridge School of Clinical Medicine, Cambridge Biomedical Campus, Cambridge, UK; 3 The Hospital for Tropical Diseases, Wellcome Trust Major Overseas Programme, Oxford University Clinical Research Unit, Ho Chi Minh City, Vietnam; 4 Aga Khan University, Karachi, Pakistan; 5 Nepal Academy of Health Sciences, Oxford University Clinical Research Unit, Kathmandu, Nepal; 6 Division of Allergy and Infectious Disease, Center for Emerging and Re-emerging Infectious Diseases, University of Washington School of Medicine, Seattle, WA, USA; 7 GSK Global Health, Tres Cantos, Madrid, Spain

## Abstract

**Background:**

Antimicrobial therapy is essential for the treatment of enteric fever, the infection caused by *Salmonella* serovars Typhi and Paratyphi A. However, an increase in resistance to key antimicrobials and the emergence of MDR and XDR in *Salmonella* Typhi poses a major threat for efficacious outpatient treatments.

**Objectives:**

We recently identified tebipenem, an oral carbapenem licensed for use for respiratory tract infections in Japan, as a potential alternative treatment for MDR/XDR *Shigella* spp. Here, we aimed to test the *in vitro* antibacterial efficacy of this drug against MDR and XDR typhoidal *Salmonella*.

**Methods:**

We determined the *in vitro* activity of tebipenem in time–kill assays against a collection of non-XDR and XDR *Salmonella* Typhi and *Salmonella* Paratyphi A (non-XDR) isolated in Nepal and Bangladesh. We also tested the efficacy of tebipenem in combination with other antimicrobials.

**Results:**

We found that both XDR and non-XDR *Salmonella* Typhi and *Salmonella* Paratyphi A are susceptible to tebipenem, exhibiting low MICs, and were killed within 8–24 h at 2–4×MIC. Additionally, tebipenem demonstrated synergy with two other antimicrobials and could efficiently induce bacterial killing.

**Conclusions:**

*Salmonella* Paratyphi A and XDR *Salmonella* Typhi display *in vitro* susceptibility to the oral carbapenem tebipenem, while synergistic activity with other antimicrobials may limit the emergence of resistance. The broad-spectrum activity of this drug against MDR/XDR organisms renders tebipenem a good candidate for clinical trials.

## Introduction

Antimicrobial resistance poses a major threat for enteric (typhoid) fever treatment, as well as infections caused by other Gram-negative bacteria, such as *Shigella* spp. and pathogenic *Escherichia coli.*^1^ Enteric fever is a life-threatening systemic disease caused by *Salmonella enterica* serovar Typhi and the various pathovars of *Salmonella* Paratyphi (A, B and C). Enteric fever remains a public health problem in many countries in South Asia and sub-Saharan Africa with poor sanitation, resulting in an estimated global incidence of >14 million cases and >135 000 deaths annually.^2^

MDR *Salmonella* Typhi (resistant to ampicillin, chloramphenicol and trimethoprim/sulfamethoxazole) have become common and been facilitated by the global expansion of the H58 lineage.^3^ More recently, XDR *Salmonella* Typhi, characterized by resistance to fluoroquinolones and third-generation cephalosporins in combination with the standard MDR phenotype, have been isolated in Pakistan.^4^ XDR *Salmonella* Typhi have since been identified in other countries and been associated with travel to Pakistan.^5^ Alarmingly, cases of XDR typhoid (identical susceptibility profile to isolates from Pakistan) with no recent travel history have recently been recorded in the USA.^6^

XDR *Salmonella* Typhi isolates remain largely susceptible to azithromycin and carbapenems,^4^^,^^7^ with guidelines in Pakistan and the American CDC recommending these antimicrobials as monotherapy or in combination for the treatment of XDR typhoid infections.^6^ However, azithromycin resistance has been recorded in both *Salmonella* Typhi and *Salmonella* Paratyphi A and appears to be increasing.^8^ The carbapenems are a potent class of β-lactam antimicrobials used to treat life-threatening bacterial infections and XDR typhoid can be effectively treated by meropenem or imipenem. Unfortunately, these antimicrobials are administered parenterally, thus largely restricting their use to inpatients.

Our repertoire of oral antimicrobials against MDR/XDR organisms is becoming limited and the emergence of XDR *Salmonella* Typhi highlights the need for alternative antimicrobials to treat infections associated with these highly resistant organisms. We recently identified tebipenem as a drug-repurposing opportunity for infections caused by MDR *Shigella*, for clinical *Shigella* isolates exhibiting MIC values of 0.02–0.15 mg/L.^9^ The prodrug, tebipenem pivoxil, is an oral carbapenem that is only licensed for use in paediatric patients with serious respiratory infections in Japan.^10^ It presents with high oral bioavailability, a broad spectrum and activation in gut enterocytes, potentially offering a solution for treating XDR infections without the requirement for hospitalization. Spero Therapeutics is developing an adult formulation with an extended half-life.^10^ The reported breakpoints for tebipenem activity against other Gram-negative bacteria, such as *Haemophilus influenza* and *Escherichia coli*, propose that tebipenem-susceptible bacteria have MIC values <1 mg/L.^10^^,^^11^ Here, we aimed to understand the potential of tebipenem as a new oral therapeutic to treat typhoid fever caused by XDR *Salmonella* Typhi.

## Materials and methods

The *Salmonella* Typhi and *Salmonella* Paratyphi A organisms used in this study were previously isolated in Nepal (*n *=* *21; non-MDR/non-XDR)^12^ and in Pakistan (*n *=* *79; all XDR).^4^ Bacteria were cultured in Mueller–Hinton (MH) medium (Sigma–Aldrich, UK) overnight at 37°C. The MIC values of tebipenem (Sigma–Aldrich, UK) were determined by an existing microdilution assay.^9^ Briefly, 10 μM tebipenem in MH broth was serially diluted and 5 × 10^5^ cfu/mL bacteria were added and incubated at 37°C overnight in a total volume of 200 μL. Bacterial growth was detected by plating 10 μL of solution from each well on Nutrient Agar (NA; Oxoid) and incubating overnight at 37°C. Results were interpreted as the minimal concentration necessary to inhibit growth (i.e. no growth visible in the 10 μL aliquot).

Time–kill curve assays were performed in 50 mL Falcon tubes by culturing *Salmonella* in MH medium in the presence of four antimicrobial concentrations in doubling dilutions ranging from 0.5×MIC to 4×MIC. Bacterial stocks were prepared in 0.9% NaCl and added to each tube to obtain a concentration of 5 × 10^5^ cfu/mL. Bacteria were grown with agitation at 200 rpm at 37°C and monitored over a time course of 24 h (0, 2, 4, 6, 8 and 24 h). For every concentration and timepoint, bacterial cultures were diluted and inoculated onto NA, before being incubated at 37°C overnight and cfu were enumerated.

Combination studies with clinical isolates were performed as previously described.^9^ MICs were determined for drug A and drug B alone and in combination. The MIC of each drug in the combination was expressed as the fraction of the MIC of the drug alone normalized to 1, representing the fractional inhibitory concentration (FIC), with the sum of the FICs [(MIC of drug A in combination/MIC of drug A alone)+(MIC of drug B in combination/MIC of drug B alone)] giving the FIC index (FICI) score.

## Results

**Figure 1.**
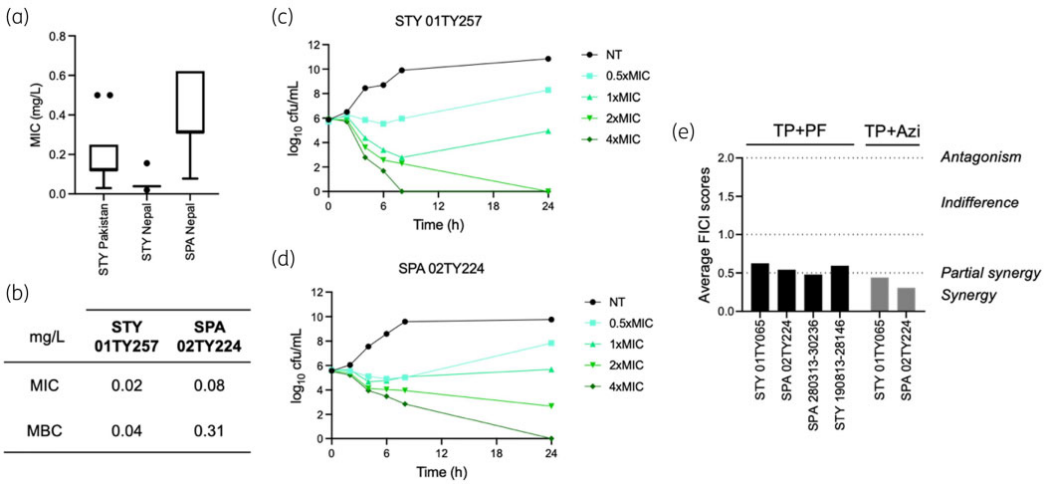
Tebipenem as an oral alternative for the treatment of enteric fever caused by MDR/XDR *Salmonella* Typhi/*Salmonella* Paratyphi A. (a) Tukey boxplots showing MIC values of tebipenem for *Salmonella* Typhi (STY) and *Salmonella* Paratyphi A (SPA) isolated from Pakistan and Nepal. The isolates from Pakistan are all XDR STY. (b) Tebipenem MIC and MBC values for STY 01TY257 and SPA 02TY224. Representative time–kill curves of STY (c) and SPA (d) isolates in various doubling concentrations of tebipenem compared with bacteria grown with no treatment (NT). (e) Bar chart showing the average FICI scores to determine the *in vitro* synergy or antagonism of tebipenem (TP) in combination with an LpxC inhibitor (PF; black) or azithromycin (Azi; grey) against various STY or SPA isolates.

To determine the repurposing potential of tebipenem for typhoidal *Salmonella*, we measured the inhibitory activity of this compound against a collection of 100 clinical non-XDR and XDR *Salmonella* Typhi and non-XDR *Salmonella* Paratyphi A from Pakistan and Nepal. The MIC values of tebipenem for tested isolates were consistently ≤0.62 mg/L (IQR = 0.12–0.25 mg/L; Figure 1a), even for the XDR isolates. The majority of *Salmonella* Typhi from both Pakistan (XDR) and Nepal (non-XDR) had lower MIC values (median = 0.12 mg/L and 0.039 mg/L, respectively) compared with Nepali *Salmonella* Paratyphi A (non-XDR) (median = 0.31 mg/L); the latter also included the least tebipenem-susceptible isolates in our collection (ED199, 02TY067, DM188 and ED293 with MICs of 0.62 mg/L). These data suggest that the drug is likely to work in enteric fever patients infected with XDR and non-XDR isolates.

Identifying that all organisms were susceptible to tebipenem, we selected two isolates (*Salmonella* Typhi 01TY257 and *Salmonella* Paratyphi A 02TY224) to further investigate the bactericidal effect of tebipenem on typhoidal *Salmonella*. The tebipenem MIC and MBC values for *Salmonella* Paratyphi A 02TY224 were 4 and 8 times higher, respectively, compared with those for *Salmonella* Typhi 01TY257 (Figure 1b). Time–kill assays of tebipenem showed that the compound exhibited high-level bactericidal activity against both isolates, with rapid killing occurring during the first 6 h of exposure (Figure 1c and d). *Salmonella* Typhi 01TY257 was effectively killed by tebipenem at 2×MIC after 24 h and at 4×MIC within 8 h (Figure 1c). In comparison, tebipenem induced complete killing of *Salmonella* Paratyphi A 02TY224 at 4×MIC only after 24 h of exposure (Figure 1d). Notably, both *Salmonella* Typhi and *Salmonella* Paratyphi A recovered growth when treated with 0.5–1×MIC after 6–8 h of tebipenem exposure (Figure 1c and d).

We next determined the synergistic abilities of tebipenem combined with azithromycin and an LpxC inhibitor (PF-5081090) in *in vitro* assays. Azithromycin often remains the last-available antimicrobial for treating uncomplicated enteric fever,^13^^,^^14^ while we have found that LpxC inhibitors show synergy with tepibenem and retain activity against *Shigella* clinical isolates.^9^ Tebipenem combined with either the LpxC inhibitor or azithromycin resulted in partial synergy (FICI scores of ≤0.5) for both tested *Salmonella* Typhi and *Salmonella* Paratyphi A isolates (Figure 1e) and notably even for azithromycin-resistant *Salmonella* Paratyphi A.

## Discussion

Carbapenems remain the last-resort treatment for many infections and thus the emergence of resistance must be mitigated. However, carbapenem resistance is not uncommon^15^ and many bacterial pathogens causing nosocomial infections, such as *Klebsiella pneumoniae*, employ resistance mechanisms, such as plasmid-borne carbapenemases and/or the modification of outer membrane influx proteins.^16^^,^^17^ Combining tebipenem with other commonly used antimicrobials with different modes of action may restore and/or increase the efficacy of both antimicrobials in a combination against MDR/XDR pathogens and may prove to be effective in reducing the risk of developing resistance to carbapenems.^9^

These results suggest that these combinations may be beneficial to protect the efficiency of tebipenem and limit the emergence of resistance to this vital class of antimicrobials. Indeed, studies with other antmicrobials targeting Gram-negative bacteria have indicated that combination therapy shows better efficacy, lower mortality, higher recovery and lower rates of resistance compared with monotherapy.^18^ Given the high *in vitro* potency of tebipenem against a range of enteric pathogens and the prodrug hydrolysis and active-ingredient release within enterocytes, we suggest it could be administered before obtaining culture results when XDR typhoid is considered.^9^ Tebipenem is already licensed in Japan (Orapenem) to treat paediatric respiratory infections and has existing safety documentation,^10^ rendering it an attractive compound for clinical trials of MDR/XDR typhoidal *Salmonella*.

We are in urgent need of new antimicrobials for the treatment of infections caused by XDR organisms and the emergence of XDR typhoid in Pakistan and the USA has left azithromycin as the only remaining oral alternative. Our data show that Orapenem (tebipenem pivoxil) may offer some respite in the community treatment of XDR enteric fever and that resistance may be prevented by combining this carbapenem with an antimicrobial with an alternative mode of action.
